# Quinia as a Stimulant to the Pregnant Uterus

**Published:** 1875-07-01

**Authors:** Albert H. Smith


### QUINIA AS A STIMULANT TO THE PREGNANT UTERUS.

By Albert H. Smith, M.D.

*From Obstetrical Journal of Great Britain and Ireland, June,* 1875.

THE active discussion carried on for several years past as to the effect of quinia upon the gravid uterus, and especially the diversity of opinion among observers, make it desirable to bring forward any facts which may tend to place the remedy in its proper position in the materia medica of obstetrics. Having for several years, and more carefully during the last few months, noticed the action of quinia during the various periods of pregnancy and parturition, and having been enabled to draw some practical conclusions of no little value to myself, I have thought that, perhaps, a brief statement of my results might be of interest to the College.

I. As to its effects upon the uterus in a quiescent state, I have, of course, only been able to make observations incidentally, giving it for the treatment of constitutional affections arising in pregnant women. But I think the experience under such circumstances is quite sufficient to establish the fact that quinia has no power in itself to excite uterine contractions. Since the subject has first been agitated, I have at many times had under care pregnant women, with disease of malarial origin, to whom I have administered quinia sulphate in doses varying from twelve to twenty grains in the twenty-four hours, without the slightest manifestation of any disturbance of the uterus; still further, I have had cases in which, with symptoms of malarial poisoning, the pregnant uterus had already become disturbed; where there were pelvic distress, tenesmus, and sacro-lumbar pain of a paroxysmal character, and where I have administered quinia in large doses, as before mentioned, with the happy result of relieving the constitutional symptoms, and at the same time of quieting the local pains. The cases could be given in detail, if time allowed; but having no points of interest, except the single facts already mentioned, it would be useless to occupy the time of the College with them.

II. As to the effect of quinia upon contractions of the uterus developed prematurely, from accidental causes, in abortions or early deliveries, my observations have been too limited to establish any positive results; but in five cases I have administered fifteen grains of the sulphate, after the process had advanced beyond the possibility of arrest, and the pains were recurring with regularity, and in none of these instances did I observe any increase in the frequency or efficiency of the contractions, or, when there was hæmorrhage, any lessening of the flow.

These observations, so far as they go, would forbid our placing quinia in the same rank with ergot, as an excito-motor stimulant with specific effect upon the uterine muscular fibre; for we know, from the experience of all observers, that ergot will not only increase the expulsive efforts of the womb when already excited, in any period of pregnancy, and in the *early* stages of hæmorrhagic abortion diminish the flow of blood by causing a condensation of the uterine fibre, and a partial closure of the open vessels from which the placenta has been detached; but that it will also throw the uterus into active contraction from a state of entire rest. In a case under my care recently, when I found it necessary to bring on labor for the expulsion of a dead fœtus, I failed to arouse the uterus to action by any of the ordinary methods in use, until I began the regular administration of ergot in full doses, when a prompt effect ensued.

III. When the uterus is in normal labor at full period of gestation, then we have quinia playing its legitimate part as an aid to parturition; and it is with special reference to its use under these conditions that I have brought the subject to your attention. For a number of years I have been in the habit of using quinia occasionally in cases of marked inertia during the stage of dilatation, in combination with other remedies, such as the administration of diffusible stimulants or hot drinks, abdominal friction, or any other means that might suggest itself in preference to the use of ergot, which, with its dangerous influences upon the child and its occasional fearful risk to the mother, and with its annoying uncertainty of action, I have long since proscribed, or at least limited, in its use during the first and second stages of labor, to rarely exceptional cases.

But for a few months past, I have been experimenting with sulphate of quinia as a promoter of normal labor from the onset of the process, and the observations, although made in a limited number of cases, show a uniformity of results which I think does not admit of the supposition of a mere coincidence. Since I began the use of the quinia, I have had under care forty-three patients of my own, besides others whom I have seen in consultation, and the experiments have been made upon forty-one of my own, aud one of those to whom I was called; of the other two cases of my own, one was a case of placenta prævia allowing no delay for experiments, while the other was completed, and the uterus in a state of firm contraction upon the placenta before I reached the patient; all of the cases to which I was called in consultation, except the one mentioned, were cases requiring forceps or other mechanical interference, in which I had nothing to do with the case before or after such aid was rendered. I mention these points to show that the forty-two cases upon which my conclusions are based were not selected, but were in the ordinary run of every-day practice. To each one of these I administered at my first visit, after actual labor-pains had begun, fifteen grains of sulphate of quinia in one dose. In every case I observed within fifteen minutes, a decided increase in the frequency and vigor of the contractions, a rapid progress of the labor, and where there was no obstruction, a speedy termination. In ten of the cases there were delays arising from malposition, from deficiency in size of the pelvis, or from disproportion of the head, requiring manipulation or instrumental aid, and all of them necessarily involving tedious dilatation. The remaining thirty-two were terminated with surprising and gratifying rapidity.

Without wishing to prolong the subject by citing the details of cases, more interesting to the observer than to others, I may mention a few typical examples.

One lady whom I saw at the request of a professional friend, was a primipara, 26 years old; had been in labor since two o'clock in the night; I saw her at twelve, noon; pains at long intervals and feeble; os only slightly patulous; no decided dilatation; strength becoming exhausted; the patient was nervous and anxious. I advised the use of sulphate of quinia fifteen grains. A few minutes after its administration a hard pain came on, followed in rapid succession by others; the os began to dilate, and in half an hour the membranes ruptured, the head coming down upon the perineum in a few more pains, and just before one o'clock, less than an hour after I had first seen the patient, the child was born. The uterus contracted firmly upon the placenta, which was removed by pressure and traction; no relaxation of the uterus or hæmorrhage more than a proper amount occurred.

A patient with her sixth child, who had had long and tedious labors previously, followed always by profuse flow, and in her last by violent flooding, had been three hours in slight pain when I first reached her; the os was just patulous, soft and flaccid, the pains making no impression in dilatation; administered the same dose of quinia as above. In a few minutes the uterus was roused into active contractions, and in half an hour the labor was completed. There was no delay in the delivery of the placenta; no disposition to hæmorrhage; on the contrary, the flow very moderate.

Another, with whom in two previous labors I had used forceps to combat the effects of inertia, had been ten hours in slow labor when I reached her; the os had but just begun to dilate; the head being forced but slightly against the rim of the cervix by the contractions. I immediately administered the full dose of quinia; the pains began in fifteen minutes to increase in frequency and intensity, and in a little over an hour the fœtus was expelled without any instrumental aid.

Other cases equally striking might be given in detail, but it would unnecessarily occupy space.

The conclusions that I have arrived at in regard to the action of the drug in these cases are as follows:

It increases the activity of the normal uterine contractions; the pains becoming more frequent and more intense, the expulsive power being greater, while the yielding of the circular fibres of the os is more prompt; the contractions maintaining their proper intermittent character, the relaxation and rest in the interval being complete; showing in this respect an entirely different action from the continuous spasmodic contraction caused by ergot. The efficiency of the contraction may be judged of from the fact, that in the thirty-two cases having no obstruction, although many were primiparæ, and a larger than usual proportion occipito-posterior positions, the average duration of active labor after the quinia was administered was about one hour. In a considerable number of the cases included, I had in several previous labors required to use forceps to combat inertia in the second stage.

It promotes permanent tonic contraction of the uterus, after the expulsion of the placenta. Several of the patients had had flooding under my care previously, some of them habitually, and some stated they had always had a profuse and weakening flow in all their other labors. In the whole forty-two I had not one case of flooding, and as a rule the uterus contracted firmly after the second stage was completed, and showed no tendency to relax afterward.

It diminishes the lochial discharge to a normal standard; many of the patients expressed surprise at the small amount of flow during the twenty-four hours following labor.

Its use is followed by less after-pains than usual in a majority of cases.

It reduces the frequency of the mother's pulse, and relieves the nervous demoralization so often seen in the first stage of labor.

Given during parturition, it never disturbs the brain or causes its usual unpleasant effects, even in patients who at other times are very susceptible to its influence. Although the dose has been uniformly fifteen grains, in only one case was the slightest sensation of cinchonism manifest, and that lasting only a moment, in a lady who knew what she had taken and was perhaps quite prepared to feel it.

Finally, I would sum up the conclusions I have adopted, perhaps hastily, though with a certain conviction of their correctness, viz.:

I. That quinia has no inherent property of stimulating the gravid uterus to contraction; being inert as to any effect upon the womb in a quiescent state, and having no decided action in accidental labors at any period of gestation.

II. That to its property as a general stimulant and promotor of vital energy and functional activity, and to that alone, is due its influence upon the uterus in normal parturition; producing then no action peculiar to itself, but merely increasing the power of the uterus to expel its contents by its own natural method, converting what is a defective or even pathological action into a simple physiological process.

III. That by availing ourselves of this power, we may, by administering full doses of sulphate of quinia at the onset of labor, favor the rapid and safe termination of what might otherwise be a tedious and exhausting work.

